# Early stage signet ring cell carcinoma of the colon examined by magnifying endoscopy with narrow-band imaging: a case report

**DOI:** 10.1186/s12876-015-0317-z

**Published:** 2015-07-24

**Authors:** Ken Ohnita, Hajime Isomoto, Taro Akashi, Keiichi Hashiguchi, Kayoko Matsushima, Hitomi Minami, Yuko Akazawa, Naoyuki Yamaguchi, Fuminao Takeshima, Kazuo To, Hiroaki Takeshita, Haruna Yasui, Kuniko Abe, Kazuhiko Nakao

**Affiliations:** 1 Department of Gastroenterology and Hepatology, Nagasaki University Hospital, 1-7-1 Sakamoto, Nagasaki, Japan; 2 First Department of Surgery, Nagasaki University Hospital, 1-7-1 Sakamoto, Nagasaki, Japan; 3 Department of Pathology, Nagasaki University Hospital, 1-7-1 Sakamoto, Nagasaki, Japan

**Keywords:** Signet ring cell carcinoma, Colon cancer, Narrow band imaging, Pit pattern

## Abstract

**Background:**

Signet ring cell carcinoma of the colon and rectum is rare, and most cases are detected at an advanced stage. We present a case of primary signet ring cell carcinoma detected at an early stage by magnifying endoscopy with narrow-band imaging (NBI) and crystal violet staining.

**Case presentation:**

A 73-year-old man visited our hospital for screening colonoscopy. Six years previously, he had undergone endoscopic submucosal dissection (ESD) for early gastric cancer. The pathological diagnosis was a well-differentiated adenocarcinoma, invading into the mucosa without lymphovascular invasion. Colonoscopy revealed a flat elevated lesion with a slightly depressed area, 20 mm in diameter, in the cecum. Further, magnifying endoscopy with NBI revealed that the surface pattern was slightly irregular and microvessels had a regular diameter and distribution in the margin of the lesion, but in the central part of the lesion, irregularity in the tumor surface pattern and form as well as in the diameter and distribution of microvessels was noted. Additionally, due to mucus, avascular areas were also observed. Magnifying endoscopy combined with 0.05 % crystal violet staining showed IIIL and V_I_ pit patterns in the margin of the lesion, and a V_I_ pit pattern in the central part of the lesion; however, due to mucus exudate, this finding could not be established with certainty. The lesion was successfully removed *en bloc* using ESD without complications. The tumor was composed mainly of signet ring cell carcinoma, partially mixed with moderately differentiated (tub2) and well-differentiated (tub1) adenocarcinomas. The tumor cells infiltrated 250 μm into the submucosal layer and involved lymphatic vessels. Therefore, the patient underwent an additional laparoscopic ileocecal resection, and the resected specimen revealed no residual carcinoma or lymph node metastasis.

**Conclusion:**

In this case report, we present a case of primary signet ring cell carcinoma detected at an early stage and identified by magnifying endoscopy with NBI and crystal violet staining.

## Background

Signet ring cell carcinoma of the colon and rectum is rare, comprising 0.1 %–2.6 % of the total cases of colorectal cancer [1]. Most cases are detected at an advanced stage and rarely at an early stage. In the diagnosis of early stage differentiated colorectal cancer, magnifying endoscopy with crystal violet staining is useful for distinguishing between malignant and benign tumors and for predicting the depth of tumor invasion [2]. Magnifying endoscopy with narrow band imaging (NBI) is also useful [3, 4], however, there has been no report on signet ring cell carcinoma of the colon observed by magnifying endoscopy with NBI. In this case report, we present a case of primary signet ring cell carcinoma detected at an early stage and examined using magnified endoscopy with NBI and crystal violet staining.

## Case presentation

**Fig. 1:**
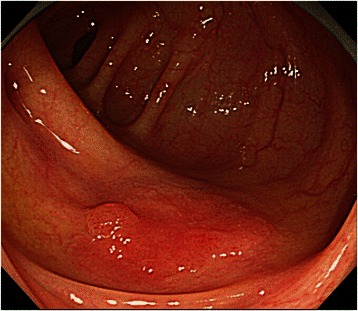
Colonoscopy revealed a flat elevated lesion in the cecum with a slightly depressed area, 20 mm in diameter

**Fig. 2:**
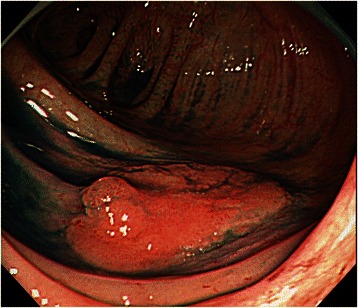
After the application of 0.2 % indigo carmine dye, the lesion could be observed more clearly

**Fig. 3:**
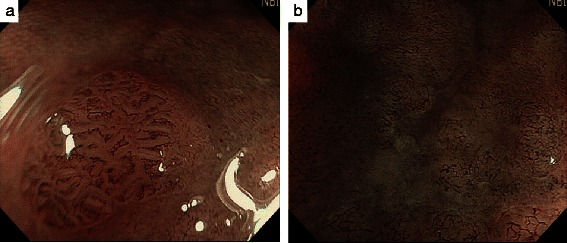
By magnifying endoscopy with narrow band imaging (NBI), the surface pattern was seen to be slightly irregular, but microvessels had a regular diameter and distribution in the margin of the lesion (**a**). However, in the central part of the lesion, irregularity in surface pattern and form as well as in the diameter and distribution of microvessels was seen; however, due to mucus, avascular areas were also seen (**b**)

**Fig. 4:**
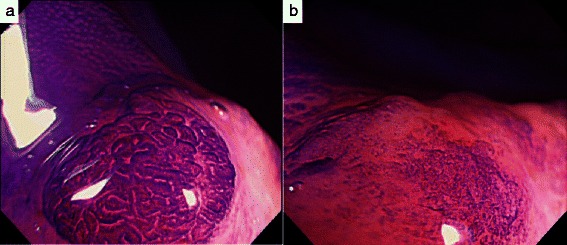
Magnifying endoscopy with 0.05 % crystal violet staining showed IIIL and V_I_ pit patterns in the margin of the lesion (**a**) and a V_I_ pit pattern in the central part (**b**; see text for details)

**Fig. 5:**
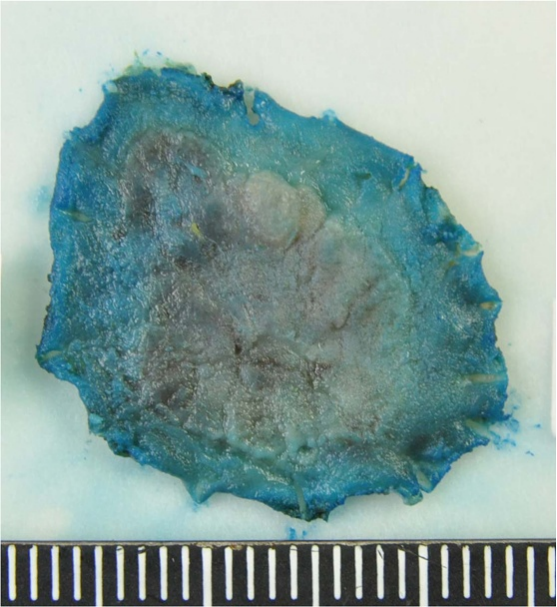
The resected specimen measured 25 × 20 mm, and the tumor measured 21 × 13 mm

**Fig. 6:**
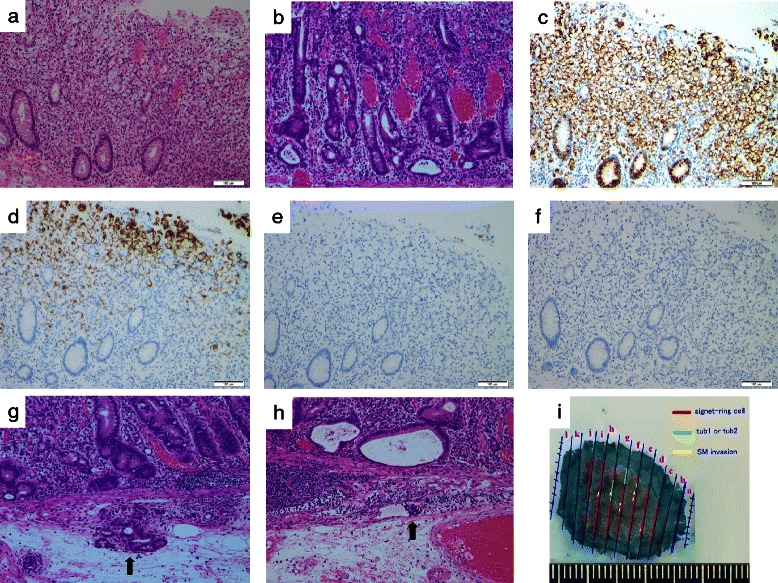
The tumor was composed of signet ring cell carcinoma (**a**), partially mixed with moderately differentiated (tub2) and well-differentiated (tub1) adenocarcinomas (**b**). In the immunostaining for the mucin phenotype, MUC2 (**c**) and MUC5AC (**d**) were positive, MUC6 (**e**) was focal positive, and MUC1 (**f**) was negative. The tumor cells infiltrated 250 μm into the submucosal layer (**g**) and invaded lymphatic vessels (**h**). The distribution of intramucosal and submucosal cancer tissue is depicted in (**i**)

A 73-year-old man with no family history of any cancer visited our hospital for screening colonoscopy. Six years ago, he had undergone endoscopic submucosal dissection (ESD) for early gastric cancer, and the pathological diagnosis was a well-differentiated adenocarcinoma extending into the mucosa, but without lymphovascular invasion. On admission, physical examination was unremarkable, and all blood tests were within the normal ranges. Colonoscopy revealed a flat elevated lesion with a slightly depressed area, 20 mm in diameter, in the cecum (Fig. 1). After the application of 0.2 % indigo carmine dye, the lesion could be observed more clearly (Fig. 2). Magnifying endoscopy with NBI revealed that the surface pattern was slightly irregular and that the microvessels were regular in terms of diameter and distribution in the margin of the lesion (Fig. 3a). In contrast, in the central part of the lesion, irregularity in the tumor surface pattern and form as well as in the diameter and distribution of microvessels was observed; however, due to mucus, avascular areas were also observed (Fig. 3b). Magnifying endoscopy with 0.05 % crystal violet staining showed IIIL and V_I_ pit patterns [2] in the margin of the lesion (Fig. 4a), while a V_I_ pit pattern was seen in the central part; however, this finding could not be fully corroborated due to the presence of mucus exudate in the central part of the lesion (Fig. 4b). In addition, abdominal computed tomography (CT) showed no lymph node or distant metastasis. We judged that the lesion was a primary adenocarcinoma, invading the mucosa and even slightly infiltrating the submucosa. After receiving written informed consent from the patient, the lesion was successfully removed en bloc using ESD without complications. The resected specimen then measured to be 25 × 20 mm, and the tumor size was 21 × 13 mm (Fig. 5). The tumor was mainly composed of signet ring cell carcinoma (Fig. 6a), partially mixed with moderately differentiated (tub2) and well-differentiated (tub1) adenocarcinomas (Fig. 6b). In the immunostaining for the mucin phenotype, MUC2 (Fig. 6c) and MUC5AC (Fig. 6d) were positive, MUC6 (Fig. 6e) was focal positive, and MUC1 (Fig. 6f) was negative. These results confirmed signet ring cell carcinoma of the colon. The tumor cells infiltrated 250 μm into the submucosal layer (Fig. 6g) and invaded lymphatic vessels (Fig. 6h), but there was no vascular invasion. The distribution of intramucosal and submucosal tumor tissue is shown in Fig. 6i. Furthermore, the patient underwent an additional laparoscopic ileocecal resection for a radical operation. The resected specimen revealed no residual carcinoma and no lymph node metastasis. At the 26-month postoperative follow-up, the patient was in good health, with no evidence of recurrence.

## Discussion

Signet ring cell carcinoma of the colon and rectum is rare; more than 96 % of cases occur in the stomach [5]. Although the present case had a history of early gastric cancer, the signet ring cell component had not been identified earlier, and no other primary lesion could be identified. Moreover, the result of the immunostaining for the mucin phenotype confirmed. Therefore, we thought that the lesion was a primary signet ring cell carcinoma of the colon. Furthermore, in the diagnosis of early stage differentiated colorectal cancer, magnifying endoscopy with crystal violet staining is useful for distinguishing between malignant and benign tumors and for predicting the depth of invasion [2]. Some studies have reported that magnifying endoscopy with NBI is also useful [3, 4], however, most colorectal cancers represent cases of differentiated adenocarcinoma, and signet ring cell carcinomas of the colon are usually detected only at an advanced stage. There are few reports on signet ring cell carcinoma of the colon examined by magnifying endoscopy. Fu et al. reported that they could not identify the pit pattern with crystal violet staining as dense mucus coated the surface of the cancer [6]. In our case, IIIL and V_I_ pit patterns were seen in the margin of the lesion, corresponding to tubular adenocarcinoma; however, because of mucus in the most of the central portion, corresponding to signet ring cell carcinoma, pit patterns could not be established with certainty. As for signet ring cell carcinoma of the stomach, we previously reported that destructive or non-structural pit patterns were often observed [7]. Because signet ring cell carcinoma produces mucus and the structure of the pits is destroyed, it is difficult to stain such lesions with crystal violet. By combining NBI with magnifying endoscopy, irregular pits surrounded by microvessels were observed in the margin of the lesion, corresponding to tubular adenocarcinoma, while irregularity of microvessels in terms of configuration, diameter, and distribution and avascular areas were also seen in the central portion, corresponding to the signet ring cell carcinoma. This may indeed indicate that signet ring cell carcinoma destroys the glandular structure. Kim et al. observed signet ring cell carcinoma of the colon using NBI and reported that the lesion could be identified clearly [8]. However, the authors did not use magnifying endoscopy. To the best of our knowledge, the present study represents the first case of signet ring cell carcinoma of the colon observed using magnifying endoscopy with NBI. There are some reports on cases of gastric signet ring cell carcinoma identified using magnifying endoscopy with NBI. Nakayoshi et al. reported that a corkscrew pattern was visible in undifferentiated gastric carcinoma [9]. In addition, it is suggested that the findings of the signet ring cell carcinoma of colon by magnifying endoscopy are similar to those of gastric signet ring cell carcinoma. In the Japanese Society for Cancer of the Colon and Rectum guidelines 2010 for the treatment of colorectal cancer, if any of the following findings are observed during the histological examination of the endoscopic resected specimen, intestinal resection with lymph node dissection should be considered as an additional surgery: (1) depth of SM invasion ≥ 1000 μm; (2) presence of vascular invasion; (3) poorly differentiated adenocarcinoma, signet-ring cell carcinoma, or mucinous carcinoma; and (4) grade 2/3 budding at the site of deepest invasion [10]. In the present case, because lymphatic vessel invasion was present and histology indicated signet ring cell carcinoma, the patient underwent an additional intestinal resection with lymph node dissection. It is not possible to diagnose vascular invasion before endoscopic resection. However, if we took a biopsy prior to endoscopic resection and confirmed the presence of signet ring cell carcinoma, the patient should have been subjected via surgery. But we didn’t doubt the presence of signet ring cell carcinoma. If we took biopsies before endoscopic resection, the lesion would be difficult to be resected endoscopically due to the fibrosis following the biopsies. Therefore, we did not take a biopsy. When we observe the similar case in future, we should take a biopsy to make a diagnosis before treatment.

## Conclusion

Although signet ring cell carcinoma of the colon is rare, it is crucial to report it to facilitate a better understanding of the diagnosis and management of this potentially life-threatening disease. We presented a case of primary signet ring cell carcinoma of the cecum detected at an early stage, removed en bloc by endoscopic submucosal dissection and subsequent laparoscopic ileocecal resection with a favorable outcome.

## Consent

Written informed consent was obtained from the patient for publication of this case report and any accompanying images.
